# Reduced retinoic acid synthesis accelerates prophase I and follicle activation

**DOI:** 10.1530/REP-20-0221

**Published:** 2020-06-08

**Authors:** Roseanne Rosario, Hazel L Stewart, Emily Walshe, Richard A Anderson

**Affiliations:** 1 MRC Centre for Reproductive Health, Queen’s Medical Research Institute, University of Edinburgh, Edinburgh, UK

## Abstract

In female mammals, reproductive potential is determined during fetal life by the formation of a non-renewable pool of primordial follicles. Initiation of meiosis is one of the defining features of germ cell differentiation and is well established to commence in response to retinoic acid. WIN 18,446 inhibits the conversion of retinol to retinoic acid, and therefore it was used to explore the impact of reduced retinoic acid synthesis on meiotic progression and thus germ cell development and subsequent primordial follicle formation. e13.5 mouse fetal ovaries were cultured *in vitro* and treated with WIN 18,446 for the first 3 days of a total of up to 12 days. Doses as low as 0.01 µM reduced transcript levels of the retinoic acid response genes *Stra8* and *Rarβ* without affecting germ cell number. Higher doses resulted in germ cell loss, rescued with the addition of retinoic acid. WIN 18,446 significantly accelerated the progression of prophase I; this was seen as early as 48 h post treatment using meiotic chromosome spreads and was still evident after 12 days of culture using Tra98/Msy2 immunostaining. Furthermore, ovaries treated with WIN 18,446 at e13.5 but not at P0 had a higher proportion of growing follicles compared to vehicle controls, thus showing evidence of increased follicle activation. These data therefore indicate that retinoic acid is not necessary for meiotic progression but may have a role in the regulation of its progression and germ cell survival at that time and provide evidence for a link between meiotic arrest and follicle growth initiation.

## Introduction

Female reproductive senescence results from the depletion of a finite ovarian follicle pool that is assembled during fetal life. These follicles are produced from primordial germ cells which transition to oocytes as they undergo loss of pluripotency, proliferate with incomplete cytokinesis forming germ cell nests, and enter into meiosis. The initiation of meiosis is one of the defining features of germ cell differentiation and occurs in fetal life in female mammals. Although meiosis is comprised of two rounds of cell division, only prophase of meiosis I occurs during fetal oogenesis, with arrest occurring before completion of the first division.

In fetal ovaries, the first signs of meiosis initiation are apparent between embryonic day 12.5 and 13.5 in mice (Adams & McLaren 2002) and from gestational week 12 in humans (Gondos *et al*. 1986, Le Bouffant *et al*. 2010). Current understanding of meiosis initiation and regulation is primarily derived from studies in mice, and it is generally accepted that the onset of meiosis depends on exposure to retinoic acid. Indeed, early studies demonstrated that long-term vitamin A deprivation (the precursor in retinoic acid synthesis) caused spermatogenic arrest in rodents (Huang & Hembree 1979, Griswold *et al.* 1989), while more recent studies have shown that maternal deficiency of vitamin A resulted in female offspring with undifferentiated germ cells which had failed to initiate meiosis, which could be rescued in pregnant vitamin A-deficient rats through administration of retinoic acid (Li & Clagett-Dame 2009). Retinoic acid is produced by the mesonephros in rodents (Bowles *et al*. 2006, Koubova *et al*. 2006) but probably by the fetal ovary itself in humans (Le Bouffant *et al*. 2010, Childs *et al*. 2011, Bowles *et al*. 2016, Frydman *et al*. 2017), through the reversible conversion of retinol (dietary vitamin A) to retinaldehyde by alcohol dehydrogenases. Retinaldehyde is then irreversibly oxidised by retinaldehyde dehydrogenases (RALDHs) 1, 2, and 3 (Griswold *et al.* 2012). In the fetal testis, endogenous retinoic acid is cleared by CYP26B1, ensuring that the onset of meiosis in male germ cells is delayed until after birth (reviewed in Feng *et al.* 2014). Due to its lipophilic nature, retinoic acid easily diffuses through cell membranes to interact with heterodimerised retinoic acid receptors (Rar α, β, and γ), which are found bound to retinoic acid-responsive elements in regulatory regions of target genes. In the presence of retinoic acid, these receptors act to enhance the transcription of genes that are otherwise silenced. Retinoic acid mediates the transcription and expression of the meiotic factor *Stra8 (stimulated by retinoic acid gene 8)*, which is required for pre-meiotic DNA replication (Baltus *et al*. 2006), and governs the subsequent events of meiotic prophase (Lin *et al.* 2008) through the upregulation of DNA meiotic recombinase *Dmc1* (Menke *et al.* 2003). In female mouse germ cells, *Rar* and *Stra8* expression have been shown to coincide with retinoic acid production and the onset of meiosis at approximately embryonic day 13.5 (Morita & Tilly 1999, Dokshin *et al*. 2013).

Following diplotene, nests of interconnected germ cells break down to release individual oocytes which associate with somatic pre-granulosa cells forming primordial follicles; this takes place during early postnatal life in rodents (Pepling & Spradling 2001) and in humans begins around 15 weeks gestation (Motta *et al.* 1997, Bendsen *et al.* 2006). These primordial follicles remain in a dormant state within the ovarian cortex until they receive signals for activation and are recruited to grow. Whether germ cell nest breakdown and primordial follicle formation are developmentally linked to meiotic progression remains poorly understood. Deletion of genes responsible for the initial stages of prophase, including *Dazl*, *Spo11*, *Dmc1*, *Atm*, *Msh4* and *Msh5*, result in loss of oocytes and an inability to form follicles, precluding analysis (Pepling 2006, Edson *et al.* 2009). Conversely, the ovaries of *Stra8*^−/−^ mice contain oocyte-like cells and follicular structures, despite being unable to initiate meiosis (Baltus *et al.* 2006, Dokshin *et al.* 2013), and depletion of *Sycp1* actually accelerates the onset of diplotene and results in primordial follicles being assembled earlier and in greater numbers than in control ovaries (Paredes *et al.* 2005). Thus the conflicting evidence surrounding the intricate relationship between meiotic progression, and particularly diplotene arrest, and primordial follicle formation needs to be explored further in physiological conditions.

WIN 18,446 belongs to the bis-dichlororacetyl-diamines (BDAD) family of compounds and can inhibit the enzymatic activity of at least one of the aldehyde dehydrogenases, thus preventing the conversion of retinol to retinoic acid (Amory *et al*. 2011). It was initially investigated as a non-hormonal male contraceptive, before being discontinued due to severe side effects in patients after alcohol consumption (Heller *et al*. 1961, Zaneveld & Waller 1989). However, when administered to male rats, WIN 18,446 caused a reduction in sperm concentration and motility without affecting Leydig cells (Brooks & van der Horst 2003), and a study in male rabbits confirmed this was likely due to reduced aldehyde dehydrogenase 1a2 activity and retinoic acid production (Amory *et al.* 2011). Although gene expression studies on WIN 18,446-cultured mouse gonads demonstrated decreased *Stra8* transcript levels after 48 h (Hogarth *et al.* 2011), no studies have investigated the impact of this inhibition on germ cell maturation and meiotic progression in the fetal ovary. Therefore, in this study, WIN 18,446 was used to explore the fate of germ cells after inhibition of retinoic acid synthesis and thus the relationship between retinoic acid, meiotic progression and primordial follicle assembly. Using a mouse fetal ovary culture model, where ovaries are dissected at e13.5 once meiosis has been initiated, we have shown that inhibition of retinoic acid synthesis with WIN 18,446 resulted in an acceleration of progression through prophase I and a subsequent increase in primordial follicle growth activation, thus suggesting a potential link between meiotic progression and primordial follicle formation and growth initiation.

## Materials and methods

### Animals

Experiments involving mice were approved by the University of Edinburgh Animal Research Ethics Committee and performed according to the UK Animal (Scientific Procedures) Act 1986. WT CD-1 mice were maintained and bred in an environmentally controlled room on a 12 h light:12 h darkness photoperiod from 0700 h each day and fed *ad libitum* according to the UK Home Office and local University of Edinburgh ethical standards. To obtain fetuses for ovary culture experiments, mouse breeding harems were set up and females checked for the presences of a copulation plug; this was designated as embryonic day 0.5 (e0.5).

### Fetal ovary culture

Pregnant timed-mated females were obtained at e13.5 and culled by cervical dislocation. Fetal ovaries with attached mesonephros were dissected from female embryos; the day of dissection was designated as day 0 of culture. Ovaries with attached mesonephros were cultured for either 2, 3 or 12 days on a 2% agar block in a 35 mm petri dish, incubated at 37°C, 5% CO_2_. For the first 3 days of culture (days 0–3), culture media contained Dulbecco’s Minimal Essential medium (Fisher Scientific, UK) supplemented with 10% fetal bovine serum, 2 mM L-glutamine, 10 μM β-mercaptoethanol and 1% sodium pyruvate. For subsequent days of culture, media were replaced with a simple culture medium consisting of α-MEM (Fisher Scientific) supplemented solely with 3 mg/mL BSA. Medium was replaced with fresh medium every 72 h. To inhibit retinoic acid production, fetal ovaries were cultured with doses of WIN 18,446 (CAS 1477-57-2, Tocris Bioscience) ranging from 0.01 µM to 5 µM or equivalent concentration of DMSO solvent for the first 3 days of culture; medium was replaced on day 3 of culture thus removing the drug. For rescue experiments, 0.7 µM of retinoic acid (Merck) was added to culture media in combination with WIN 18,446.

### Newborn ovary culture

Ovaries were dissected from postnatal day 0 (P0) mice and cultured on floating polycarbonate membranes (Whatman, Sigma-Aldrich) in 24-well plates containing α-MEM (Fisher Scientific) supplemented with 3 mg/mL BSA, incubated at 37°C, 5% CO_2_. To investigate an independent effect on follicle activation, ovaries were cultured with 1 µM of WIN 18,446 for 3 days. Subsequently, ovaries were transferred to drug-free medium, which was replaced every 48 h. Ovaries were cultured for a total of 6 days.

### RNA extraction, cDNA synthesis and RT-qPCR

To assess the expression of retinoic acid response genes, cultured mouse fetal ovaries were collected in TRIzol (Fisher Scientific), homogenised using a motorised pellet pestle, and RNA was extracted using the RNeasy mini kit (Qiagen) according to manufacturer’s instructions. RNA was reverse transcribed to cDNA using concentrated random primers and Superscript III reverse transcriptase (Fisher Scientific) according to manufacturer’s instructions, and the cDNA synthesis reaction was diluted appropriately before proceeding. Primers for quantitative RT-PCR (RT-qPCR) were designed to amplify all transcript variants and are exon-spanning. Primer pair efficiencies were calculated with the LinReg PCR applet (Ramakers *et al.* 2003). Each reaction was performed in a final volume of 10 µL, with 1× Brilliant III SYBR Green qPCR Master Mix (Agilent), 20 pmol of each primer and 2 µL of diluted cDNA. Primer sequences are as follows written in the 5′ to 3′ direction:* Stra8* F GTTTCCTGCGTGTTCCACAAG, *Stra8* R CACCCGAGGCTCAAGCTTC, *Rarβ* F TGTTTACCTGTTCACAAGCCA, *Rarβ* R GGAGGAGACCGGAACAAGTT, *Dazl* F tggaccgaagcatacagaca, *Dazl* R actgcccgacttcttctgaa, *Sycp3* F cagagccagagaatgaaagca, *Sycp3* R atttgccatctcttgctgct, and see van den Bergen *et al.* 2009 for *Mapk1 (Mitogen-activated protein kinase 1)* and *Canx (Calnexin precursor)* sequences. Each cDNA sample was analysed in triplicate. Target genes were normalised to the geometric mean expression of *Canx and Mapk1* (van den Bergen *et al*. 2009). Data analysis for relative quantification of gene expression and calculation of s.d. was performed as outlined (Livak & Schmittgen 2001, Vandesompele *et al*. 2002).

### Immunohistochemistry

Single immunostaining for Tra98 (germ cell specific antigen) was used to identify germ cells for counting after 3 days of culture with WIN 18,446. Double immunostaining for Msy2 and Tra98 was used to assess oocyte maturation on day 12 of culture (ovaries only exposed to WIN 18,446 for first 3 days of culture). Cultured fetal ovaries were fixed for 2 h in Bouins solution. After processing, tissue was sectioned at 5 µm, dewaxed, rehydrated and antigen retrieval carried out in 0.01 M citrate buffer pH 6. Endogenous peroxidase activity was blocked with Dako REAL Peroxidase-Blocking solution (Agilent). Tissues were blocked in PBS containing 5% BSA and 20% normal goat serum and then incubated overnight at 4°C with primary antibodies diluted in blocking serum (Tra98: 1 in 200 (Ab82527, Abcam), Msy2: 1 in 400 (Ab82527, Abcam)). Single immunostaining for Tra98 was carried out using ImmPRESS reagent (Vector Laboratories, UK) according to manufacturer’s instructions, and slides were counterstained with hematoxylin before mounting. For double immunofluorescence, tissues were incubated with a peroxidase-conjugated antibody for 30 min at room temperature, before visualisation using Tyramide Signal Amplification (TSA) (PerkinElmer) according to manufacturer’s instruction. The first primary antibody (Msy2) was then removed by a second antigen retrieval performed by microwaving slides in boiling 0.01 M citrate buffer for 2.5 min. After cooling, slides were blocked as before and incubated overnight at 4°C with anti-rat Tra98 (1 in 400). Secondary antibody and visualisation steps were carried out as mentioned previously, before counterstaining with DAPI and mounting. Images were captured using an Axioscan slide scanner (Carl Zeiss) and a 710 LSM confocal microscope (Carl Zeiss) with Zen 2009 software.

### Chromosome spreads and immunostaining

Ovaries were taken at day 2 of culture (that is 48 h post culture setup), and for each *n*, five ovaries were pooled to ensure enough oocytes for quantification and oocyte chromosome spreads were prepared as described previously (Peters *et al*. 1997). For immunostaining, slides were first washed in PBS, then blocked in PBS containing 0.15% BSA, 0.1% Tween 20 and 5% goat serum. Slides were then incubated with mouse anti-Sycp3 (1:500. Abcam, ab97672), rabbit anti-Rpa (1:300, He *et al*. 1995) and guinea pig anti-Sycp1 (1:200, Bolcun-Filas *et al*. 2009) diluted in block buffer. Alexa Fluor-conjugated secondary antibodies (Invitrogen) were used at a 1:500 dilution, and 2 ng/μL DAPI was used to fluorescently stain DNA before mounting. Images were captured using a Zeiss Imager Z1 fluorescence microscope with Plan-neofluar objectives (Carl Zeiss), and image capture was performed using Zen 2009 software.

### Meiotic staging

Oocytes were staged, blinded to treatment, as outlined in Crichton *et al*. 2017, 2018. Briefly, staging was based on synaptonemal complex formation/dissolution, using staining patterns of the axial element protein Sycp3, which marks the axis of each homologue, and the transverse filament protein Sycp1, which marks regions of chromosome synapsis. All Sycp3-positive oocytes were scored. Leptotene nuclei contained fragmented axial elements and a lack of synapsis indicated by the absence of co-localisation between Sycp3 and the transverse filament marker Sycp1. Zygotene nuclei were identified by extensive axial element formation and partial synapsis. Pachytene oocytes contained at least one fully synapsed pair of homologous chromosomes. Recombination foci marked by Rpa were used to distinguish between zygotene and diplotene, with Rpa foci being absent in diplotene nuclei (Shi *et al*. 2019); no diplotene nuclei were observed.

### Germ cell counts and histological follicle assessment

Fetal (e13.5) ovaries were fixed for 2 h in Bouins solution on days 3 and 12 of culture, and newborn (p0) ovaries were fixed for 2 h in Bouins solution on day 6 of culture, paraffin wax-embedded, sectioned and stained for Tra98 (germ cell marker) or with hematoxylin and eosin. Assessment of germ cell number was carried out on 5-µM thick tissue sections and every sixth section, spanning the entire volume of the ovary (6–10 sections, depending on tissue orientation), with the Abercrombie correction factor being applied to raw counts to estimate total germ cell number per ovary (Abercrombie 1946). Assessment of follicle stage and health was carried out (blinded) on every sixth section of day 12 of e13.5 cultured ovaries or day 6 of P0 cultured ovaries. A follicle was counted only where the analysed section contained an oocyte with a visible germinal vesicle. Primordial follicles (PMF) were defined as having only squamous pre-granulosa cells, transitionary follicles (TRNS) had both squamous and cuboidal granulosa cells, and primary follicles (PRIM) had a complete layer of cuboidal granulosa cells. Follicle health was assessed as described previously (Stefansdottir *et al*. 2016).

### Statistical analysis

All data are shown as mean ±s.e.m. and were analysed using GraphPad Prism 7 software (GraphPad Software Inc.). Mann–Whitney *U*-test and Kruskal-Wallis test statistics were carried out as appropriate. A *P* value of <0.05 was considered statistically significant.

## Results

### WIN 18,446 inhibits the expression of retinoic acid response genes *Stra8* and *Rarb* in mouse fetal ovaries

**Figure 1:**
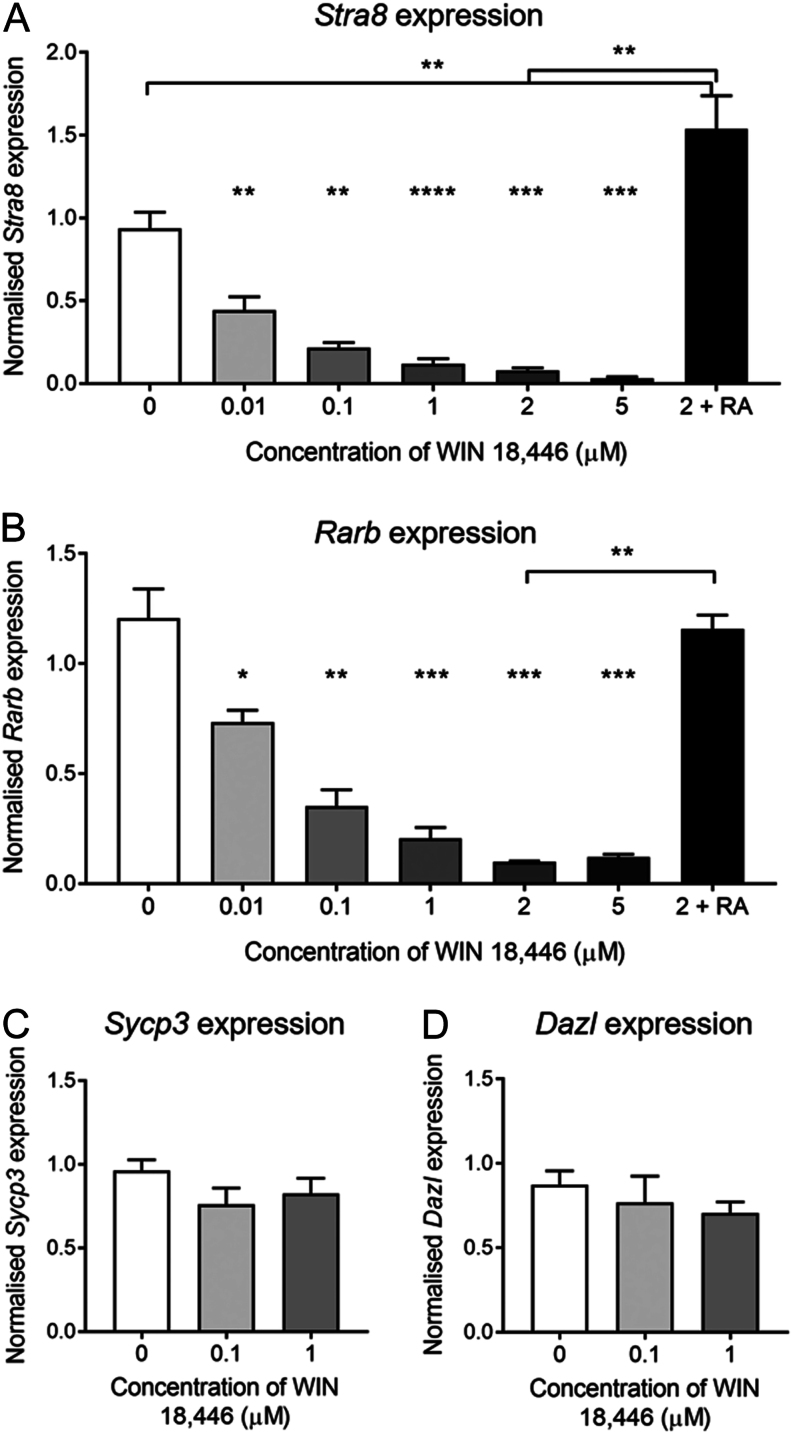
Retinoic acid response gene expression in e13.5 mouse ovaries cultured for 2 days with WIN 18,446 or vehicle control. (A) *Stra8*, (B) *Rarβ*, (C) *Sycp3* and (D) *Dazl* mRNA expression in ovaries cultured for 2 days with WIN 18,446 doses ranging from 0.01 µM to 5 µM, 2 µM WIN 18,446 with 0.7 µM of retinoic acid, or vehicle control. Graph displays mean of *n* = 5 ovaries for each group. Error bars indicate ± s.e.m. *P < 0.016; **P < 0.008; ***P < 0.0007.

To examine the effect of WIN 18,446 on the fetal ovary, ovaries were isolated from e13.5 mice and cultured with doses of WIN 18,446 ranging from 0.01 µM to 5 µM or vehicle only. RT-qPCR was used to evaluate the expression of retinoic acid response genes *Stra8* and *Rarb* on day 2 of culture (Fig. 1). Culture with the lowest dose of WIN 18,446 (0.01µM) resulted in approximately 50% reduction in the transcript levels of *Stra8* (*P* = 0.008), and a clear dose response was observed with the 5 µM dose, causing approximately 97% reduction in *Stra8* expression (Fig. 1A). To demonstrate that this reduction was caused by reduced retinoic acid synthesis, fetal ovaries were cultured with 0.7 µM of retinoic acid in combination with 2 µM of WIN 18,446; this stimulated *Stra8* expression at least 20-fold when compared to ovaries cultured with 2 µM of WIN 18,446 alone (*P* = 0.016). Furthermore, the addition of retinoic acid increased *Stra8* expression 0.5 fold above what was observed in vehicle-cultured ovaries (*P* = 0.014). *Rarb* expression was also reduced by WIN 18,446 with a similar dose response (Fig. 1B). The addition of retinoic acid increased *Rarb* transcript levels compared to ovaries cultured with 2 µM of WIN 18,446 only (*P* = 0.008), to a similar level of expression in vehicle-cultured ovaries (Fig. 1B). However, culture with WIN 18,446 did not have any significant impact on the expression of *Sycp3* and *Dazl* in e13.5 cultured ovaries (Fig. 1C and D); both genes are important for meiotic initiation and progression but are not considered to be direct targets of retinoic acid. These gene expression data indicate that treatment with WIN 18,446 is sufficient to prevent retinoic acid synthesis by blocking the conversion of retinol to retinoic acid in the mouse fetal ovary, as previously demonstrated.

### WIN 18,446 causes germ cell loss at 2 µM but not at 0.1 µM and 1 µM

**Figure 2:**
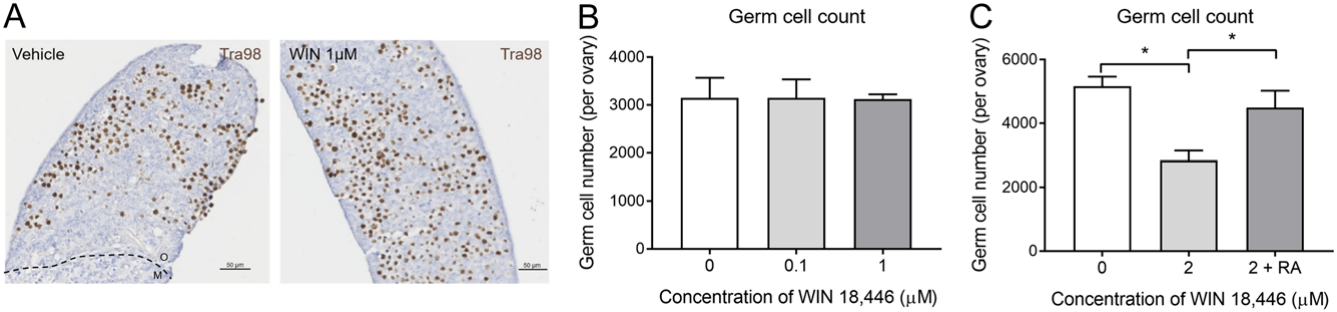
Germ cell number in e13.5 mouse ovaries cultured for 3 days with WIN 18,446 or vehicle control. (A) Representative image of Tra98 immunostaining used to quantify germ cells in vehicle-control and WIN 18,446 ovaries cultured for 3 days. Dashed line denotes border between mesonephros (M) and ovary (O). Scale bar = 50 µM. (B) Germ cell number was quantified after 3 days of culture with 0.1 µM and 1 µM of WIN 18,446 or vehicle control. Error bars indicate ± s.e.m. *n* = 5 ovaries per group. (C) Germ cell number was quantified after 3 days of culture with 2 µM WIN 18,446, 2 µM WIN 18,446 + 0.7 µM retinoic acid or vehicle control. Error bars indicate ± s.e.m. *n* = 5 ovaries per group. *P = 0.029.

Given the dramatic changes in *Stra8* and *Rarb* expression following culture with WIN 18,446, we sought to identify whether, in part, this may have been caused by a reduction in germ cell number. Immunostaining for the germ cell marker Tra98 was utilised to facilitate germ cell quantification after 3 days of culture with WIN 18,446 (Fig. 2A), and we chose to investigate the three mid-range doses 0.1, 1 and 2 µM, as these resulted in >50% reduction in the expression of *Stra8* and *Rarb*. There was no significant difference in total germ cell number per ovary between ovaries cultured with WIN 18,446 at 0.1 or 1 µM and vehicle controls (Fig. 2B). Therefore, this finding confirms that the significant reduction in *Stra8* and *Rarb* transcript levels at these lower doses depicted in Fig. 1 is due to the action of WIN 18,446 inhibiting retinoic acid synthesis rather than decreased germ cell number. Treatment with 2 µM WIN 18,446, however, resulted in a reduction in germ cell number by approximately 40%; this loss was prevented by the addition of 0.7 µM retinoic acid (Fig. 2C), suggesting that retinoic acid may have a role in germ cell survival.

### Reduced retinoic acid synthesis accelerates oocyte progression through meiotic prophase I in cultured mouse fetal ovaries

**Figure 3:**
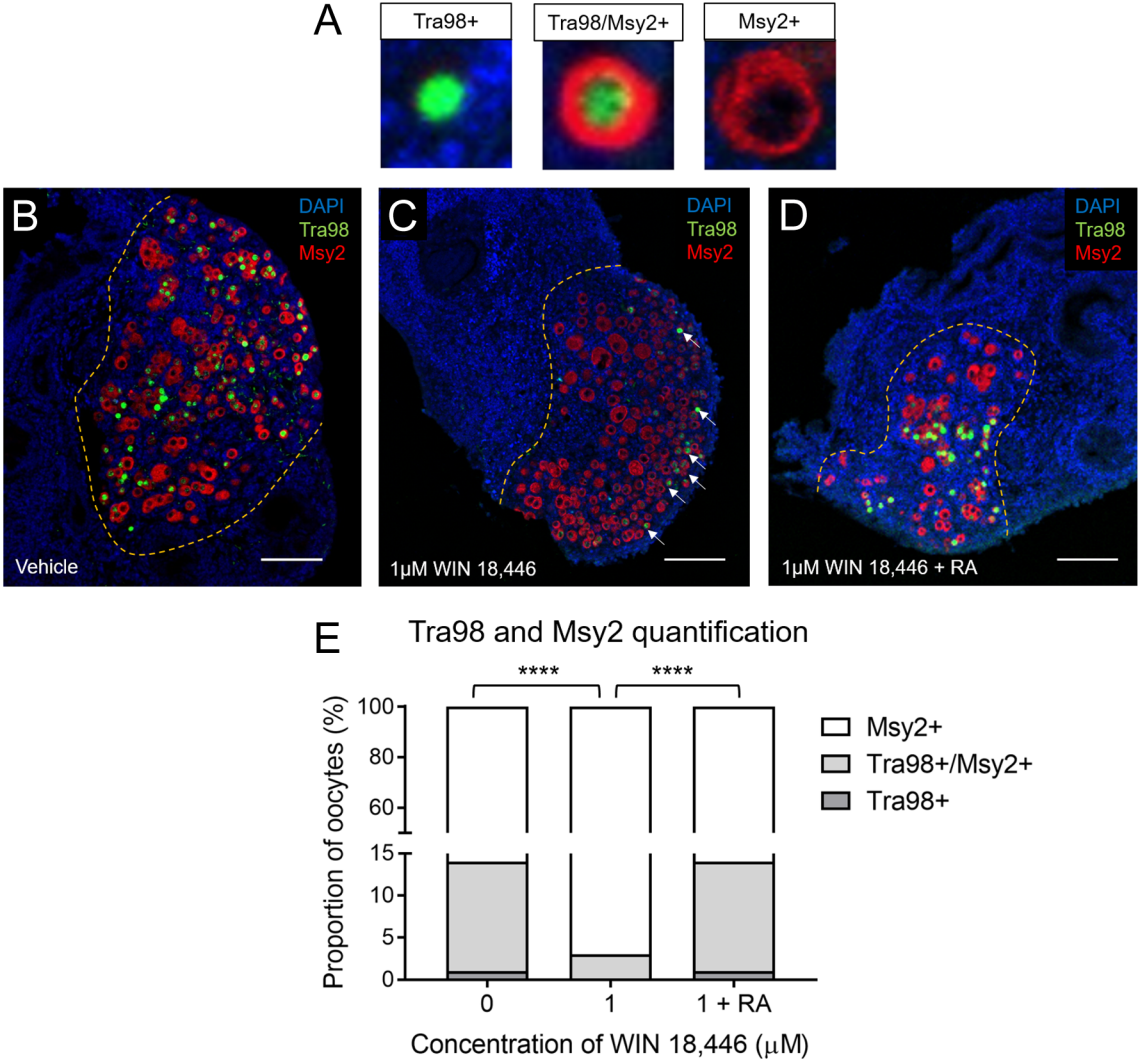
Tra98 and Msy2 double immunostaining on e13.5 mouse ovaries cultured for 12 days with WIN 18,446 or vehicle control. (A, B, C and D) Representative images of Tra98 (green) and Msy2 (red) immunostaining used to quantify germ cells in ovaries treated with vehicle-control, 1 µM WIN 18,446, and 1 µM WIN 18,446 + 0.7 µM RA and cultured for 12 days. Yellow dashed line denotes border between mesonephros and ovary. Scale bar = 100 µM. (E) Quantification of Tra98 and Msy2 immunostaining in vehicle-control, 1 µM WIN 18,446 and 1 µM WIN 18,446 + 0.7 µM retinoic acid ovaries cultured for 12 days. Error bars indicate ± s.e.m. *n* = 5 ovaries per group. ****P < 0.0001.

Double immunostaining for Tra98 and Msy2 as differential molecular markers of meiotic progression was carried out to ascertain the effect of WIN 18,446 on oocyte progression through prophase I. This experiment was carried out using only the 0.1 µM and 1 µM doses of WIN 18,446, given that these doses caused >50% reduction in the expression of *Stra8* and *Rarb* without any germ cell loss. Immunostaining was performed on ovaries cultured for 12 days, a time-frame which spans the onset of meiosis in germ cells to the formation of primordial follicles and the subsequent initiation of growth in some follicles. Tra98 is expressed in the nucleus of germ cells from their arrival at the gonadal ridge up until arrest at dictyate, when oocytes acquire a layer of granulosa cells (Enders & May 1994), while Msy2 expression in the oocyte cytoplasm correlates with arrival at the diplotene stage (though it is not a marker for diplotene arrest *per se*) (Burks *et al.* 2019) (Fig. 3A). Given the proposed importance of retinoic acid in initiating meiosis, we hypothesised that culture with 1 µM of WIN 18,446 would impede meiotic progression; however, we observed that there were more Tra98 positive oocytes in vehicle-control-cultured ovaries compared with WIN 18,446-cultured ovaries (Fig. 3B and C). Quantification of oocytes as Tra98 positive (including those that co-expressed Msy2) or Tra98 negative showed that 14.2% (±3.6%) of oocytes expressed Tra98 in vehicle cultured-ovaries, while only 3.2% (±1.7%) of oocytes were Tra98 positive in ovaries cultured with 1 µM of WIN 18,446 (*P* <0.0001) (Fig. 3E). Culture with 1 µM of WIN 18,446 and 0.7 µM of retinoic acid rescued this phenotype, with the proportion of Tra98 positive oocytes (14 ± 2.1%) returning to what was observed in vehicle-cultured ovaries (*P* < 0.0001 vs WIN 18,446 only) (Fig. 3D and E). Again, a clear dose response was observed, as ovaries cultured with 0.1 µM of WIN 18,446 had proportions of Tra98-positive and Msy2-positive oocytes that were in between vehicle-cultured ovaries and 1 µM WIN 18,446-cultured ovaries (data not shown). With regards to location of these oocytes, there appeared to be no obvious pattern in distribution of Tra98-positive and Msy2-positive within vehicle-control-cultured ovaries; however, oocytes positive for Tra98 only and both Tra98 and Msy2 were more frequently located on the periphery of WIN 18,446-cultured ovaries (highlighted by arrows in Fig. 3C). Taken together, these data suggest that culture with WIN 18,446 actually accelerates progression through prophase I, as ovaries cultured with WIN 18,446 had significantly fewer Tra98 positive oocytes than vehicle cultured-ovaries.

**Figure 4:**
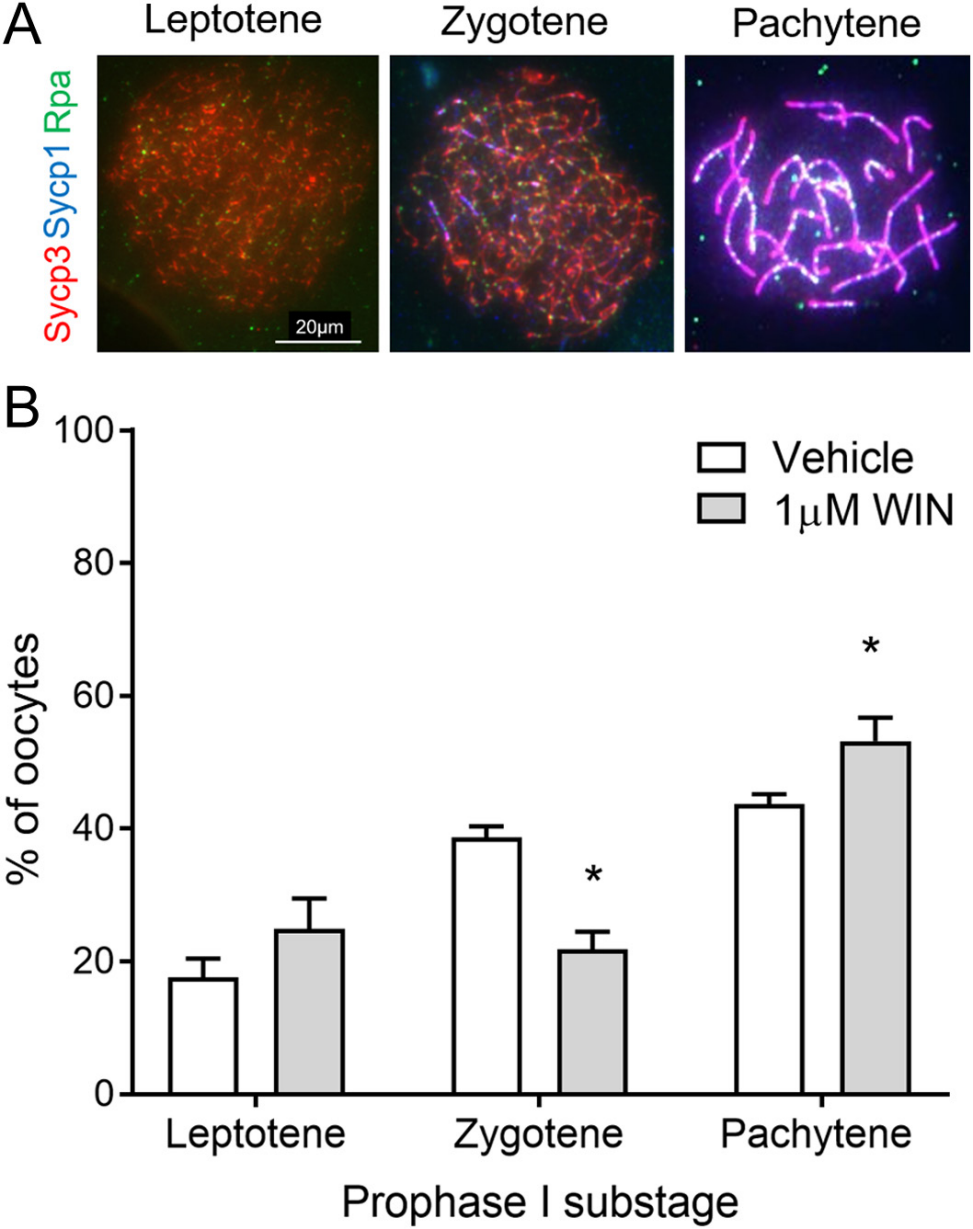
Sub-staging meiotic prophase I oocytes from e13.5 mouse ovaries cultured for 2 days with WIN 18,446 or vehicle control. (A) Immunostaining of Sycp3 (red), Sycp1 (blue) and Rpa (green) to assess axial element and transverse filament formation, respectively. Representative merged images displayed. Scale bar = 20 µM. (B) Relative proportions of prophase I substages from ovaries cultured for 2 days with vehicle-control and 1 µM WIN 18,446. Graph displays mean percentages from four experiment repeats, and a total of 258 oocytes from vehicle cultured-ovaries and 250 from WIN 18,446 cultured ovaries were analysed. Error bars indicate ± s.e.m. *P < 0.04.

As the Tra98/Msy2 quantification data was collected at the end of the culture period, we investigated whether this accelerated progression could be observed at an earlier point in culture. Oocyte chromosome spreads were therefore prepared from ovaries on day 2 of culture to capture oocytes in early meiotic prophase I. For sub-staging, oocytes were stained with antibodies against components of the synaptonemal complex: axial element protein Sycp3, and transverse filament protein Sycp1, as well as the recombination protein Rpa. A total of 258 oocytes from vehicle cultured-ovaries and 250 from ovaries cultured with 1 µM WIN 18,446 were analysed, blinded to the treatment group, using this dose only to maximise likely effects. Oocytes at three different stages were identified: leptotene, with short fragments of axial element but no synapsis; zygotene, with nuclei containing some regions of axial element undergoing synapsis, but this was not complete along the axial length; and pachytene, which had at least one pair of fully synapsed chromosomes. No diplotene oocytes were observed. Rpa recombination foci were present at all stages identified, but were less prevalent in pachytene oocytes (Fig. 4A).

In vehicle-cultured ovaries, an average of 17.6% (±2.9) of oocytes were in leptotene, 38.7% (±1.7) in zygotene, and 43.7% (±1.5) in pachytene (Fig. 4B). While there was no significant difference in the proportion of leptotene oocytes, representation of the different substages in ovaries cultured with WIN 18,446 showed a slight skew toward the latter stages of prophase I, with a significant decrease in the proportion of zygotene oocytes compared to vehicle-cultured ovaries (*P* = 0.029) and a significant increase in the proportion of pachytene oocytes (*P* = 0.029). In WIN 18,446-cultured ovaries, on average 25.0% (±4.7) of oocytes were in leptotene, 21.9% (±2.6) in zygotene, and 53.2% (±3.5) in pachytene (Fig. 4B). These data demonstrate that the acceleration of meiosis seen on day 12 of culture caused by reduced retinoic acid synthesis as a result of culture with WIN 18,446 can be observed as early as 48 h after treatment.

### Reduced retinoic acid synthesis accelerates follicle activation in culture mouse fetal ovaries

**Figure 5:**
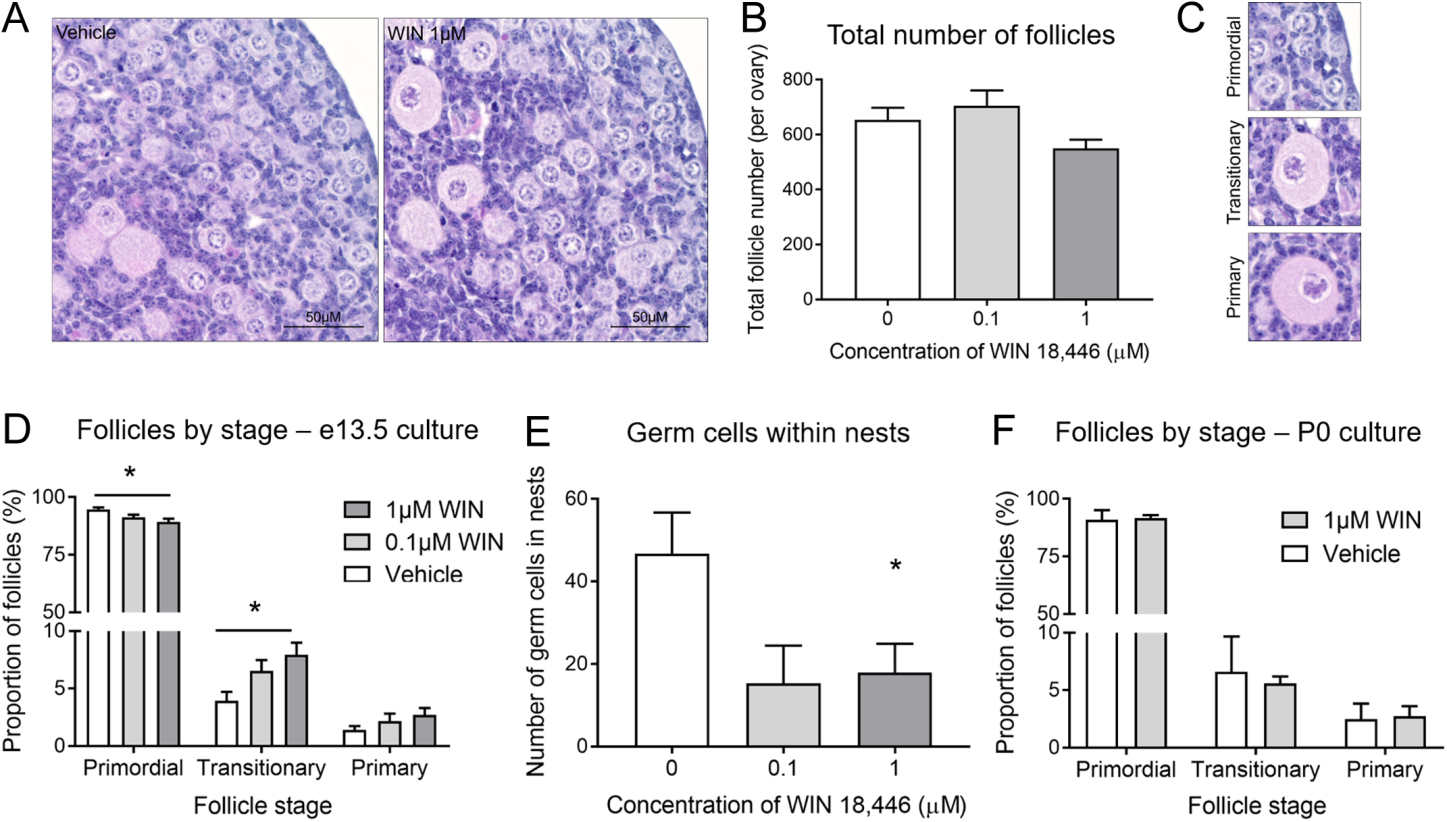
Assessment of follicles from e13.5 mouse ovaries cultured for 12 days with WIN 18,446 or vehicle control (A) Representative hematoxylin and eosin images from vehicle-control and 1 µM WIN 18,446 e13.5 ovaries cultured for 12 days. Scale bars = 50 µM. (B) Graph depicts total number of follicles in vehicle-control and WIN 18,446 e13.5 ovaries cultured for 12 days. Error bars indicate ± s.e.m. *n* = 6 ovaries per group. (C) Representative hematoxylin and eosin images of primordial (PMF), transitionary (TRNS) and primary (PRIM) follicles. Primordial follicles (PMF) were defined as having only squamous pre-granulosa cells, transitionary follicles (TRNS) were considered to have both squamous and cuboidal granulosa cells, and primary follicles (PRIM) had a complete layer of cuboidal granulosa cells. (D) Graph depicts the proportion of follicles staged as PMF, TRNS or PRIM in vehicle-control and WIN 18,446 e13.5 ovaries cultured for 12 days. Error bars indicate ± s.e.m. *n* = 6 ovaries per group. *P* < 0.001. (E) Graph depicts number of germ cells found in nest structures in vehicle-control and WIN 18,446 e13.5 ovaries cultured for 12 days. All germ cells found in nests in each ovary were counted. Error bars indicate ± s.e.m. *n* = 6 ovaries per group, *P* = 0.05 (F) Graph depicts the proportion of follicles staged as PMF, TRNS or PRIM in vehicle-control and 1 µM WIN 18,446 P0 ovaries cultured for 6 days. Error bars indicate ± s.e.m. *n* = 4 ovaries per group.

As culture with WIN 18,446 accelerated oocyte progression through prophase I, we explored the impact of this acceleration on primordial follicle formation and subsequent follicle growth initiation, using 0.1 µM and 1 µM doses of WIN 18,446, as these had no effect on germ cell number in earlier experiments. At day 12 of culture, the total number of follicles present in vehicle and WIN 18,446-cultured ovaries was assessed and follicles were staged as primordial, transitionary or primary. There was no significant difference in the total follicle number between vehicle-cultured ovaries and ovaries cultured with either 0.1 µM or 1 µM of WIN 18,446 (Fig. 5A and B). There was also no difference in follicle health post culture with WIN 18,446, as approximately 4% of all follicles in each treatment group were unhealthy. However, ovaries cultured with both doses of WIN 18,446 had proportionally fewer primordial follicles and more transitionary follicles compared with vehicle-cultured ovaries (*P* = 0.032 and *P* = 0.040, respectively) (Fig. 5C and D). Ovaries cultured with WIN 18,446 also had fewer oocytes within germ cell nest structures, suggesting these structures had broken down to form primordial follicles earlier than germ cell nests in vehicle-cultured ovaries, although this was only significant with 1 µM of WIN 18,446 (*P* = 0.05; 0.01 µM dose *P* = 0.06) (Fig. 5E). As the evidence of follicle activation was greatest using 1 µM of WIN 18,446, we sought to investigate whether this dose would have a similar effect on follicle growth in P0 ovaries, that is, independently of an effect on meiotic progression. There was no indication of increased follicle growth when P0 ovaries were cultured with 1 µM of WIN 18,446 (Fig. 5F). Thus, these data suggest that the advanced completion of meiotic prophase in oocytes after treatment with WIN 18,446 results in an increased rate of initial follicle growth activation, with more primordial follicles in WIN 18,446 treated ovaries initiating growth than in their control counterparts.

## Discussion

This present study, to our knowledge, is the first in-depth investigation into the impact of inhibition of retinoic acid synthesis on meiotic progression in the fetal ovary and its relationship with subsequent follicle formation and growth initiation. We have used the compound WIN 18,446 to inhibit retinoic acid synthesis in a fetal mouse ovary culture system that supports germ cell survival and progression through prophase I of meiosis up to diplotene arrest, followed by primordial follicle formation and subsequent growth initiation (Stefansdottir *et al*. 2016, Rosario *et al.* 2019). Fetal ovaries are cultured from e13.5, a stage when germ cells are committed to oocyte development and enter the first stages of meiosis (Adams & McLaren 2002); thus, in this study we are investigating the role of retinoic acid on meiotic progression, distinct from its initiation. Previous studies have demonstrated that treatment with WIN 18,446 results in a vitamin A-deficiency phenotype in the testes of adult male rabbits (Amory *et al*. 2011), and experiments in adult male mice (Brooks & van der Horst 2003) and in cultured testes from adult, neonatal and embryonic mice have shown a reduction in the levels of tissue retinoic acid and *Stra8* transcripts with treatment with WIN 18,446 (Hogarth *et al*. 2011). However, there has been little research into the effects of this compound on the ovary and female fertility, with only one study reporting decreased *Stra8* expression in e11.5 mouse ovaries cultured for 48 h with 1 µM WIN 18,446 (Hogarth *et al.* 2011). We have confirmed and furthered this finding by demonstrating that WIN 18,446 concentrations as low as 0.01 µM are sufficient to cause at least 50% reduction in *Stra8* and *Rarb* expression in e13.5 mouse fetal ovaries. Importantly, we have also demonstrated this altered gene expression is not due to germ cell loss, not investigated in the previous study. While WIN 18,446 doses up to 1 µM were not damaging, the germ cell loss evident at higher doses of WIN 18,446 could be rescued with the addition of retinoic acid, highlighting the potential of retinoic acid as a germ cell survival factor; however, this notion needs further exploration.

A growing body of evidence implicates retinoic acid, with the subsequent transcription of *Stra8*, as a key requirement to drive the initiation of meiosis in both male and female mammals. This has resulted in a mechanistic model involving the interplay of retinoic acid, enzymes involved in its synthesis and the receptors to which it binds, and in the prepubertal testis, protection against its actions by metabolising enzymes (Feng *et al.* 2014). It is important to note that much of this work does not detail the involvement of retinoic acid once meiosis is underway. However, conversely to expectations based on this model, after retinoic acid inhibition in our culture system, germ cells appear to have progressed through stages of prophase I faster than germ cells in vehicle-control-cultured ovaries. This acceleration was evident as early as 48 h in culture, with chromosome spread data from vehicle-control-cultured ovaries having a lower proportion of zygotene oocytes (39% vs 22% after WIN 18,446 treatment), while WIN 18,446-cultured ovaries had many oocytes in pachytene (53% vs 44% in vehicle controls). This result is in contrast to findings in vitamin A deficient rat embryonic ovaries, where, at e18.5, vitamin A deficiency had no impact on germ cell number but *Stra8* induction was absent and most germ cells were unable to enter meiosis, as assessed by a lack of Sycp3 staining usually found at the onset of leptotene (Li & Clagett-Dame 2009). Furthermore, although germ cells should have entered meiosis by e18.5, the expression of the pluripotency marker Oct4 persisted; the commitment of ovarian germ cells to meiosis is normally associated with the downregulation of *Oct4* (Pesce *et al*. 1998). Vitamin A deprivation in male rodents also results in spermatogenic arrest (Morales & Griswold 1987, Griswold *et al.* 1989, van Pelt & de Rooij 1991, Li *et al*. 2011); however, large doses of retinoic acid can induce resumption of meiosis in male and female vitamin A deficiency models, suggesting that it is retinoic acid and not its precursor retinol that is the active factor (van Pelt & de Rooij 1991, Li & Clagett-Dame 2009). Given that these data are from models where retinoic acid levels were reduced from conception, it would appear that timing of retinoic acid inhibition may be key in determining the subsequent effects on meiotic onset and progression; as germ cells have already initiated meiosis in our e13.5 culture model, we are able to identify potential effects on meiotic progression/germ cell development that are distinct from the need for retinoic acid for initiation of meiosis. Indeed, culture of mouse fetal ovaries at e11.5 with the synthetic retinoic acid receptor antagonist BMS has the expected effect of blocking meiosis initiation (Minkina *et al*. 2017).

Accelerated progression through various stages of meiosis I and II has been previously reported. Specifically related to prophase I, deletion of the kinase Kin-18 or checkpoint protein Pch-2 in C. elegans leads to accelerated prophase I progression, aberrant oocyte development, and ectopic apoptosis (Deshong *et al*. 2014, Yin *et al*. 2016). In chickens, exposure to bisphenol A, an endocrine-disrupting chemical with weak oestrogenic activity, has been reported to affect the formation of primordial follicles by promoting meiotic progression of oocytes via estrogen receptor beta signaling pathways (Yu *et al*. 2018). However, the most relevant phenotype is that of Sycp1 depletion in e21 rat fetal ovaries, which resulted in a decrease and increase in Tra98 and Mys2 expressing oocytes, respectively (Paredes *et al*. 2005). While those authors concluded that expression of Msy2 reflected attainment of diplotene, it has recently been demonstrated that it is not an exact marker of diplotene *per se* as demonstrated via chromosome spreads, although it does correlate with arrival at the diplotene stage (Burks *et al.* 2019). Nonetheless, using this Tra98/Msy2 immunostaining strategy we too observed a reduced proportion of Tra98 positive oocytes in ovaries where retinoic acid synthesis was blocked by WIN 18,446, and this effect was rescued with the addition of retinoic acid. As Tra98 is found in the nucleus of germ cells prior to dictyate arrest (it is lost from spermatocytes in late pachytene and diplotene and thought to be lost from oocytes at the same time (Enders & May 1994)), it would appear that the reduction of retinoic acid caused an acceleration of germ cell maturation.

In addition to accelerated prophase progression, Paredes* et al*. also observed increased folliculogenesis in Sycp1-deficient ovaries, with primordial follicles assembled earlier and in greater numbers than in control ovaries and more primary follicles indicating increased initiation of follicle growth (Paredes *et al*. 2005). Although we did not observe any difference in total follicle number after retinoic acid inhibition, we did see evidence of earlier oocyte nest breakdown and a modest increase in follicle activation in WIN 18,446-cultured ovaries; that is, these ovaries had proportionally fewer primordial and more transitionary follicles compared with vehicle-control-cultured ovaries. Interestingly, when we cultured newborn P0 ovaries with WIN 18,446 for the same duration (3 days exposure), we did not observe any increase in follicle growth. This is unlike the effect of growth factors such as FGF (Nilsson & Skinner 2004) and PDGF (Nilsson *et al*. 2006), which have been shown to increase levels of Kit-ligand (Parrott & Skinner 1999) and enhance the transition of primordial follicles to primary stage in newborn rat ovaries. Therefore, this would indicate that the alterations here identified in follicle activation after culture with WIN 18,446 at e13.5 are related to events taking place within germ cells during the first 3 days of culture. Furthermore, as WIN 18,446 is removed from the culture medium after this, it would seem these early changes in germ cells not only effect meiotic progression but also create knock-on effects for primordial follicle formation and growth initiation. From our data, one may speculate that the effects on meiotic progression and follicle activation may even be linked, as suggested by the ‘production line hypothesis’. According to this hypothesis, germ cells in female mammals become committed to meiosis and enter prophase sequentially in fetal life and the oocytes thus generated are activated to grow in the same sequence as that of meiotic entry (Polani & Crolla 1991, Hirshfield 1992, Mork *et al*. 2012), although findings in human and mouse fetal oocytes suggest otherwise (Speed & Chandley 1983, Rowsey *et al*. 2014). It is worth noting that our experiments do not exclude the impact of reduced retinoic acid synthesis on the surrounding somatic environment and the indirect effect this could have on germ cell maturation, given the bidirectional communication between these two cell types. Indeed, we have previously demonstrated the expression of retinoic acid receptors in pre-granulosa cells of the human fetal ovary (Childs *et al.* 2011). However, deletion of all three retinoic acid receptors in the fetal mouse ovary at the time of sex determination did not significantly affect ovarian differentiation, follicle development or female fertility, thus suggesting that retinoic acid signaling is dispensable for somatic development and function in the mouse ovary (Minkina *et al*. 2017).

In conclusion, the present data demonstrate that WIN 18,446 is highly effective at reducing retinoic acid synthesis in the mouse fetal ovary, enabling investigation into how retinoic acid regulates germ cell development after the initiation of meiosis in females. We have shown that reduced retinoic acid synthesis resulted in accelerated meiotic prophase I progression and increased follicle growth activation in mouse ovaries cultured at e13.5. These data thus suggest the importance of retinoic acid not just in the onset of meiosis but in germ cell survival and subsequent meiotic progression and support a link between meiosis and follicle formation.

## Declaration of interest

The authors declare that there is no conflict of interest that could be perceived as prejudicing the impartiality of the research reported.

## Funding

The authors’ work in this field is supported by grants from the Medical Research Council (G1100357 to R A A, MR/N022556/1 to the MRC Centre for Reproductive Health) and the Biotechnology and Biological Sciences Research Council (BB/R015635/1 to R A A and R R).

## Author contribution statement

R R and R A A designed the experiments. R R, H L S and E W carried out experiments. R R wrote the manuscript. All authors contributed to data interpretation, editing the manuscript, and its final approval.
